# Synthesis and evaluation of antitumor activities of novel chiral 1,2,4-triazole Schiff bases bearing γ-butenolide moiety

**DOI:** 10.1186/2191-2858-2-26

**Published:** 2012-07-03

**Authors:** Xiang Li, Xue-Qiang Li, He-Mei Liu, Xue-Zhang Zhou, Zhi-Hui Shao

**Affiliations:** 1 Key Laboratory of Energy Sources & Chemical Engineering, Development Center of Natural Products and Medication and School of Chemistry and Chemical Engineering, Ningxia University, Yinchuan, 750021, China; 2 Key Lab of Ministry of Education for Protection and Utilization of Special Biological Resources in Western China, School of Life Science, Ningxia University, Yinchuan, 750021, China; 3 Key Laboratory of Medicinal Chemistry for Natural Resource (Yunnan University), Ministry of Education, Yunnan University, Kunming, 650091, China

**Keywords:** 1,2,4-triazole, Schiff base, γ-butenolide, A activity, HeLa cells

## Abstract

**Background:**

1,2,4-Triazole derivatives have received much attention due to their versatile biological properties including antibacterial, antifungal, anticonvulsant, antiinflammatory, anticancer, and antiproliferative properties. 1,2,4-Triazole nucleus has been incorporated into a wide variety of therapeutically interesting molecules to transform them into better drugs. Schiff bases of 1,2,4-triazoles have also been found to possess extensive biological activities. On the other hand, γ-substituted butenolide moiety represents a biological important entity that is present in numerous biologically active natural products.

**Results:**

We have described herein the synthesis of 12 hybrid 1,2,4-triazole Schiff bases bearing γ-substituted butenolide moiety. These compounds were synthesized by utilizing the tandem asymmetric Michael addition/elimination reaction as the key step. All the new compounds were evaluated for their *in vitro* anticancer activity.

**Conclusions:**

Tandem asymmetric Michael addition/elimination approach has offered an easy access to new chiral 1,2,4-triazole compounds **7a**-**7l**. All these chiral 1,2,4-triazole derivatives exhibited good anticancer activities towards Hela. Of all the tested compounds, the chiral compound **7l** with an IC_50_ of 1.8 μM was found to be the most active.

## Background

Cancer, a diverse group of diseases characterized by the proliferation and spread of abnormal cells, is a major worldwide problem. Therefore, the discovery and development of new potent and selective anticancer drugs are of high importance in modern cancer research.

1,2,4-Triazole derivatives have received much attention due to their versatile biological properties including antibacterial, antifungal, anticonvulsant, antiinflammatory, anticancer, and antiproliferative properties [1-10]. 1,2,4-Triazole nucleus has been incorporated into a wide variety of therapeutically interesting molecules to transform them into better drugs [11-13]. Schiff bases of 1,2,4-triazoles have also been found to possess extensive biological activities [14-18]. On the other hand, γ-substituted butenolide moiety represents a biological important entity that is present in numerous biologically active natural products [19-24].

**Figure 1:**
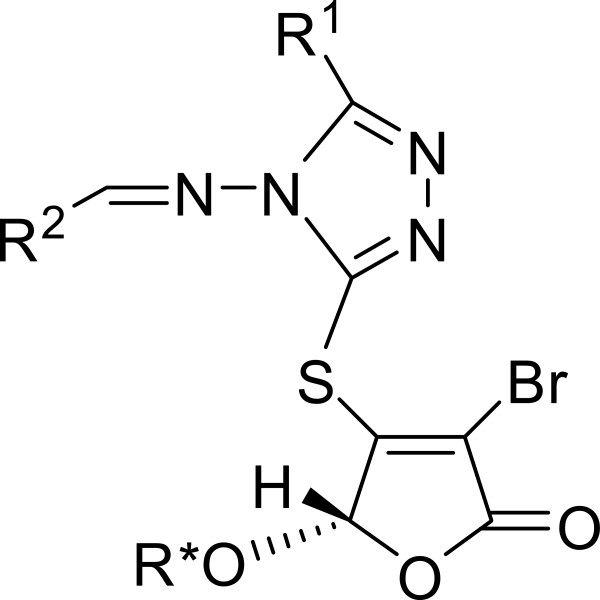
The general structure of target compounds.

Recently, we reported on the synthesis of a series of hybrid 1,3,4-thiadiazoles derivatives possessing γ-substituted butenolide moiety, which exhibited good anticancer activities against cervical cancer cells [25]. In continuation of our studies on the identification of potential active antitumor compounds, herein we report the synthesis and evaluation of a new series of hybrid 1,2,4-triazole Schiff bases bearing γ-substituted butenolide moiety as potential anticancer agents (Figure 1). To the best of authors’ knowledge, the synthesis and anticancer activities of this types of compounds have not been reported so far.

## Results and discussion

The enantiomerically pure γ-substituted butenolides **1** were synthesized via acetalization of mucobromic acid by employing (−)-menthol and (+)-borneol as a chiral auxiliary, respectively, and followed by resolution of the resulting diastereomers [25-27].

**Scheme 1:**
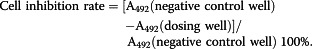
Synthesis of 1,2,4-triazole Schiff bases 6.

The 1,2,4-triazole Schiff bases **6** were synthesized by condensation 4-amino-5-substituted-4*H*-1,2,4-triazol-3-thiols **5** with aromatic aldehydes in glacial acetic acid (Scheme 1) [14]. The 4-amino-5-substituted-4*H*-1,2,4-triazol-3-thiols **5** were prepared according to the previous procedure [28,29]. When R^1^ is methyl, the compound **5a** was prepared by heating a mixture of thiocarbohydrazide with acetic acid [28]. When R^1^ are aryl, a different procedure was employed as aromatic carboxylic acids are generally solid, have high melting points, and are difficult to react with thiocarbohydrazide fully [29]. Thus, staring from aromatic carboxylic acid esters **2**, the aroyl hydrazides **3** were obtained by reaction with hydrazine in EtOH. Treatment of the aroyl hydrazides **3** with CS_2_ under a basic condition (KOH/EtOH) gave the corresponding potassium aroyl dithiocarbazates **4**. Then, the resulting compounds **4** were cyclized with hydrazine to provide the compounds **5b–d** in good yields.

**Scheme 2:**
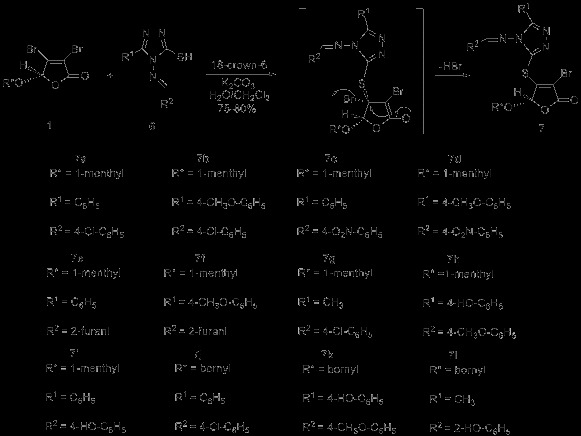
Synthesis of target compounds 7a–l.

The target compounds **7a–l** were prepared via tandem Michael addition–elimination reaction of γ-substituted butenolides **1** with 5-substituted 1,2,4-triazole Schiff bases **6** under phase-transfer catalysis conditions (Scheme 2).

**Figure 2:**
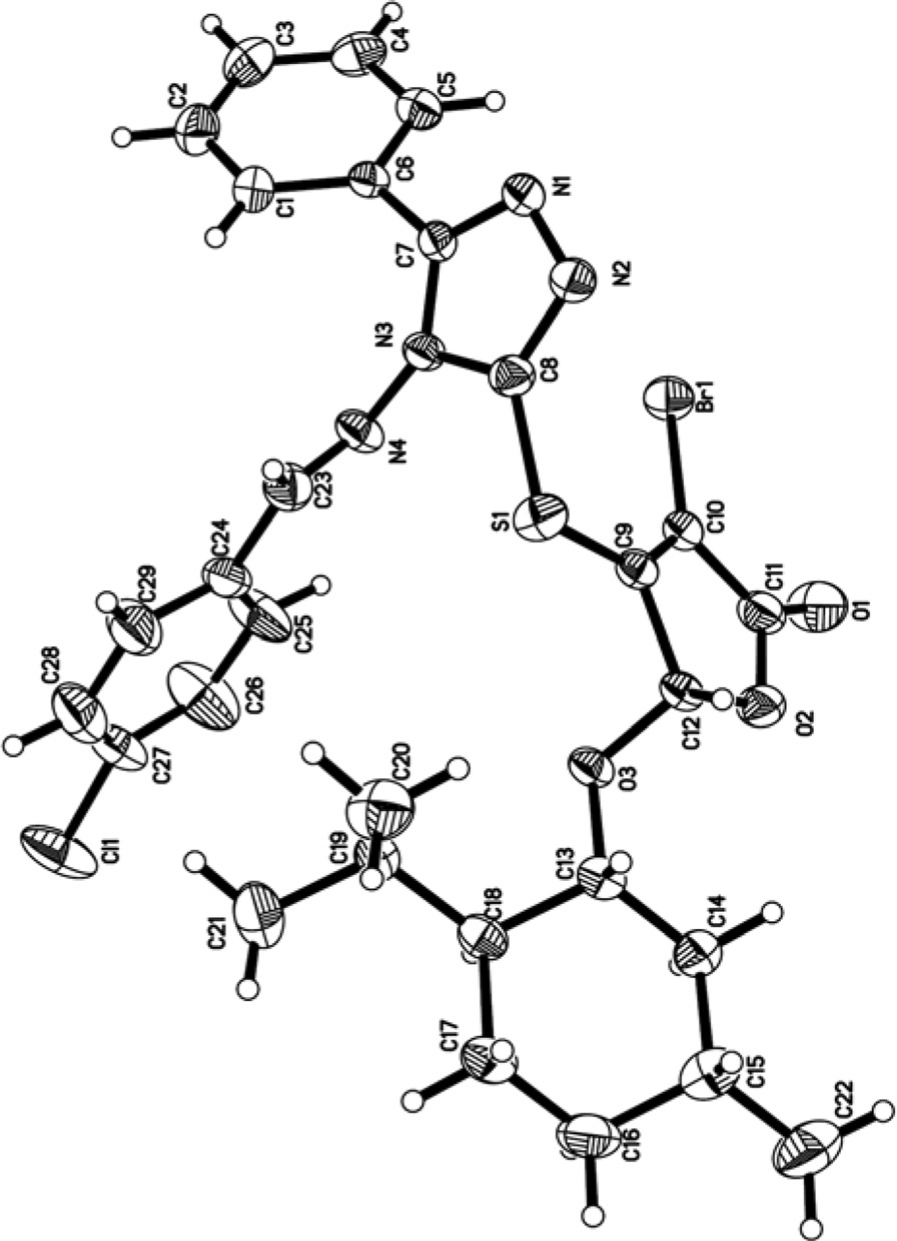
ORTEP view of the crystal structure of compound 7a.

The structures of these new compounds **7a–l** were characterized with IR, ^1^H, ^13^ C NMR, and LC-MS spectra. In addition, the molecular structure of **7a** was unambiguously confirmed through X-ray crystallography (Figure 2).^a^

All newly synthesized compounds **7a–l** were initially evaluated for their *in vitro* anticancer activities against cervical cancer cell lines (HeLa) using the MTT assay, and the results were summarized in Table 1. All the compounds **7a**–**l** displayed good inhibition activities on HeLa cell lines. Of all the studied compounds, the compound **7l** exhibited the best inhibitory activity with an IC_50_ of 1.8 μM.

**Table 1:** ***In vitro*anticancer activities against HeLa cell lines with compounds 7a–l (*n*= 3)**

**Compound**	**IC**_**50**_**(μM)**	**Compound**	**IC**_**50**_**(μM)**
**7a**	19.7	**7h**	7.1
**7b**	4.4	**7i**	3.7
**7c**	11.6	**7j**	4.5
**7d**	11.2	**7k**	6.2
**7e**	6.8	**7l**	**1.8**
**7f**	5.1	DDP (Cisplatin)	2.6
**7g**	8.2		

The IC_50_ values represent the compound concentration (μM) required to inhibit tumor cell proliferation by 50%.

Then, the growth inhibition rates of HeLa cell lines with compounds **7a–l** at different concentrations (0.1–20 μM) were evaluated (Table 2). After being treated with 20 μg/mL compound **7l** for 24 h, the growth inhibition rate was the highest (90.0%).

**Table 2:** Growth inhibition rates of HeLa cell lines with compounds 7a–l at different concentrations

**Compounds**			**Inhibition rates (%)**		
	**1.25 μM**	**2.5 μM**	**5 μM**	**10 μM**	**20 μM**
**7a**	1.2	8.7	16.1	30.2	41.9
**7b**	26.2	30.2	53.8	65.2	85.7
**7c**	9.4	35.3	21.3	52.3	60.2
**7d**	17.9	11.0	34.8	47.7	65.5
**7e**	10.9	11.4	24.3	76.0	85.2
**7f**	10.3	27.4	56.9	73.4	85.1
**7g**	18.0	30.9	43.4	49.7	67.1
**7h**	8.3	14.3	40.7	77.5	71.7
**7i**	14.3	35.6	67.8	85.4	87.7
**7j**	24.0	32.5	54.1	67.6	81.2
**7k**	14.8	36.2	60.0	56.7	67.3
**7l**	47.5	45.4	74.1	88.6	90.0

## Experimental

All the chemicals were used as-received without further purification unless otherwise stated. IR spectra were recorded on a FTIR-8400S spectrometer as KBr disks. ^1^H NMR and ^13^ C NMR spectra were obtained with a Bruker Avance III 400 MHz spectrometer in chloroform-d (CDCl_3_) and tetramethylsilane was used as an internal standard. Diffraction measurement was made on a Bruker AXS SMART 1000 CCD diffractometer with graphite-monochromatized Mo Kα radiation (λ = 0.71073 Å). All the melting points were determined on a WRS-1B digital melting point apparatus and are uncorrected. Thin-layer chromatography (TLC) was carried out on silica GF254 plates (Qingdao Haiyang Chemical Co., Ltd., China).

### General procedure for the synthesis of compounds 7

To an aqueous solution of dichloromethane was sequentially added the compounds **1** (1.0 mmol), potassium carbonate (1.0 mmol), 18-crown-6 (0.1 mmol), and the compounds **6** (1.1 mmol). The resulting mixture was stirred at room temperature, and the reaction was monitored by TLC. On completion of the reaction (10–20 h), the mixture was exacted and the organic layer was washed with saturated brine. Then the organic layer was dried over anhydrous MgSO_4_, filtered, and concentrated *in vacuo* The purification of the residue by silica gel column chromatography or crystallizations yielded the desired compounds **7a-l** in 65-89% yields (For the characterization of compound **7a**-**7l**, please see the Additional file 1: Supporting Information). Compound **7 l**: white solid, 76% yield, [α]_D_^20^ = −37.2 (*c* = 0.5 M, CHCl_3_). mp 131–132°C. IR (KBr) 3210, 1780, 1603, 1523, 1440, 1421, 1319, 1212, 1134, 993 cm^-1^. ^1^H NMR (400 MHz, CDCl_3_) 10.04 (s, 1H), 8.73 (s, 1H), 7.59-7.04 (m, 2H), 7.14-7.06 (m, 2H), 6.20 (s, 1H), 3.81 (m, 1H), 2.59 (s, 3H), 2.25-2.22 (m, 1H), 1.69-1.09 (m, 6H), 0.78-0.74 (m, 6H), 0.53 (s, 3H). ^13^ C NMR (100 MHz, CDCl_3_) 170.4, 164.0, 160.3, 152.9, 151.0, 138.2, 136.3, 133.7, 120.6, 118.1, 115.4, 112.8, 103.1, 88.8, 49.3, 47.6, 44.7, 36.7, 27.9, 26.5, 19.5, 18.7, 13.3, 11.2. HRMS calcd. for C_24_H_27_Br N_4_O_4_S [M]^+^: 546.0936, found 546.0933.

### Pharmacology

Cells (1 × 10^4^ in 100 μL) were seeded on 96-well plates in triplicate. Following a 24-h culture at 37°C, the medium was replaced with fresh medium at various concentrations (1.25, 2.5, 5, 10, 20 μg/mL) of compounds **7a–l** in a final volume of 110 μL. At the same time, set drug-free medium negative control well, and solvent control well of the same volume of dimethyl sulfoxide (DMSO). Cells were incubated at 37°C for 24 h. Then, 20 μL of 3-(4,5-dimethylthiazol-2-yl)-2,5-diphenyltetrazolium bromide (MTT) (2 mg/mL in a phosphate buffer solution) was added to each well, incubated for an additional 4 h, the plates were centrifuged at 1000 r/min for 10 min, then the medium was removed. MTT formazan precipitates were dissolved in 100 μL of DMSO, shaken mechanically for 10 min and then read immediately at 492 nm in a plate reader (Opsys MR, Denex Technology, USA).

$$\begin{array}{ll} \text{Cell inhibition rate} & =\left[{\text{A}}_{492},\left(\text{negative control well}\right)\right)\\ & \;-\left({\text{A}}_{492},\left(\text{dosing well}\right)\right]/\\ & \;{\text{A}}_{492}\left(\text{negative control well}\right)100\%\text{.} \end{array}$$

## Conclusions

In summary, a new type of chiral 1,2,4-triazole Schiff bases bearing γ-substituted butenolide moiety have been synthesized and their *in vitro* anticancer activities against have been evaluated. These chiral 1,2,4-triazole derivatives exhibited good anticancer activities towards HeLa. The compound **7l** with an IC_50_ of 1.8 μM was found to be the most active. Further studies of anticancer activities of these compounds are in progress in our group.

## Endnote

^a^The molecular structure of the product **7a** was determined by means of X-ray crystallographic studies. CCDC 829447 (**7a**) contains the supplementary crystallographic data for this article. These data can be obtained free of charge from The Cambridge Crystallographic Data Centre via http://www.ccdc.cam.ac.uk/data_request/cif.

## Competing interests

The authors declare that they have no competing interests.

## Authors’ contributions

ZS and XL carried out the design of the project, and drafted the manuscript. XL and HL synthesized target compounds. XZ evaluated in vitro anticancer activities against cervical cancer cell lines (HeLa). All authors read and approved the final manuscript.

## Supporting information available

Experimental procedures, spectral data of new compounds.

## Supplementary Material

Additional file 1 **Supporting Information Available.** Experimental procedures, spectral data of new compounds. Click here for file
