# Supplementary material for: Synthesis and evaluation of antitumor activities of novel chiral 1,2,4-triazole Schiff bases bearing γ-butenolide moiety
Source: Org Med Chem Lett. 2012 Jul 3;2:26. doi: 10.1186/2191-2858-2-26 (PMC3490741; doi:10.1186/2191-2858-2-26)
Supplement: Additional file 1 — Supporting Information Available. Experimental procedures, spectral data of new compounds. [file 2191-2858-2-26-S1.doc]

Supporting Information

**Synthesis and evaluation of antitumor activities of novel chiral 1,2,4-triazole Schiff bases bearing γ-butenolide moiety**

Xiang Li^1,3^, Xue-Qiang Li^1^*, He-Mei Liu^1^, Xue-Zhang Zhou^2^ and Zhi-Hui Shao^3^*

^1^ Key Laboratory of Energy Sources ＆ Chemical Engineering, Development Center of Natural Products and Medication and School of Chemistry and Chemical Engineering, Ningxia University , Yinchuan 750021, China

^2^ Key Lab of Ministry of Education for Protection and Utilization of Special Biological Resources in Western China, School of Life Science, Ningxia University, Yinchuan 750021, China

^3^ Key Laboratory of Medicinal Chemistry for Natural Resource (Yunnan University), Ministry of Education, Yunnan University, Kunming 650091, China

* Correspondence: zhihui_shao@hotmail.com (Z. Shao), [lixq@nxu.edu.cn](mailto:lixq@nxu.edu.cn) (X. Li)

**1. General**

Thin-layer chromatography (TLC) was carried out on silica GF254 plates (Qingdao Haiyang Chemical Co., Ltd, China). All the melting points were determined on a WRS-1B digital melting point apparatus and are uncorrected. IR spectra were recorded on a FTIR-8400S spectrometer as KBr discs. ^1^H NMR and ^13^C NMR spectra were obtained with a Bruker Avance III 400 MHz spectrometer in chloroform-d (CDCl_3_) and tetramethylsilane (TMS) was used as an internal standard. Diffraction measurement was made on a Bruker AXS SMART 1000 CCD diffractometer with graphite-monochromatized Mo Kα radiation (*λ* = 0.71073 Å). All chemicals were used as received without further purification unless otherwise stated.

**2. General procedure for the synthesis of compounds 7**

To an aqueous solution of dichloromethane was sequentially added the compounds **1** (1.0 mmol), potassium carbonate (1.0 mmol), 18-crown-6 (0.1 mmol), and the compounds **6** (1.1 mmol). The resulting mixture was stirred at room temperature, and the reaction was monitored by TLC. On completion of the reaction (10-20 h), the mixture was exacted and the organic layer was washed with saturated brine. Then the organic layer was dried over anhydrous MgSO_4_, filtered, and concentrated *in vacuo* The purification of the residue by silica gel column chromatography or crystallizations yielded the desired compounds **7a-l** in 65-89% yields.

Compound **7a**: 83% yield. [α]_D_^20^ = -45.4 (*c* = 0.5 M, CHCl_3_). mp = 191–192 ^o^C. IR (KBr): 2956, 1778 1595, 1565, 1524, 1454 cm-^1^. ^1^H NMR (400 MHz, CDCl_3_): *δ* = 8.42 (s, 1H) ,7.85-7.87 (m, 2H) ,7.73-7.84 (m, 3H), 7.46-7.52 (m, 4H), 6.45 (s, 1H), 3.53 (1H, ddd, *J* = 4.4, 4.4, 4.4 Hz), 2.26-2.22 (m, 1H), 1.95-1.89 (m, 1H), 1.61-1.56 (m, 2H), 1.37-1.35 (m, 1H), 1.20-0.99 (m, 2H), 0.90 (d, *J* = 6.8 Hz, 3H), 0.88-0.78 (m, 2H), 0.76 (d, *J* = 6.8 Hz, 3H), 0.53 (d, *J* = 6.8 Hz, 3H). ^13^C NMR (100 MHz, CDCl_3_): *δ* = 165.9, 164.4, 152.5, 152.0, 140.5, 140.4, 130.8, 130.4, 129.9, 129.4, 129.1, 128.3, 125.7, 114.5, 102.7, 82.9 , 48.1 , 42.1, 33.9 , 31.5 , 25.3, 22.8 , 22.1 , 21.1, 15.7. MS (ESI): 629.1 [M]^+^, 631.0 [M+2]^+^.

Compound **7b**: 87% yield. [α]_D_^20^ = -90.5 (*c* = 0.5 M, CHCl_3_). mp = 183-185 ^o^C. IR (KBr): 2956, 1784, 1610, 1594, 1475, 1439, 1316, 1256, 1177, 1090, 968 cm-^1^. ^1^H NMR (400 MHz, CDCl_3_): *δ* = 8.48 (s,1H), 7.85-7.76 (m, 4H), 7.54-7.50(m, 2H), 7.00-6.98 (m, 2H), 6.42 (s, 1H), 3.86 (s, 3H), 3.52 (ddd, *J* = 4.4, 4.4, 4.4Hz, 1H), 2.17-2.14 (m, 1H), 1.90-1.86 (m, 1H), 1.62-1.54 (m, 2H), 1.37-1.35 (m, 1H), 1.25-0.99 (m, 2H), 0.90 (d, *J* = 6.4Hz, 3H), 0.89-0.78 (m, 2H), 0.76 (d , *J* = 7.2Hz, 3H), 0.54 d, *J* = 6.8Hz, 3H). ^13^C NMR (100 MHz, CDCl_3_): *δ* = 165.8, 164.5, 161.5, 152.7, 152.1, 140.4, 139.6, 130.4, 129.9, 129.8, 129.5, 118.05, 114.5, 114.2, 102.6, 82.8, 55.4, 48.1, 42.1, 33.9, 31.5, 25.3, 22.8, 22.1, 21.1, 15.7. MS (ESI): 659.1 [M]^+^, 661.0 [M+2]^+^.

Compound **7c**: 77% yield. [α]_D_^20^ = -32.9 (*c* = 0.5 M, CHCl_3_). mp = 166-167 ^o^C. IR (KBr): 2955, 1774, 1593, 1524, 1471, 1451, 1346, 1316, 1215, 1134, 968 cm-^1^. ^1^H NMR (400 MHz, CDCl_3_): *δ* = 8.59 (s, 1H), 8.39-8.37 (m, 2H), 8.01-7.83 (m, 4H), 7.66-7.49 (m, 3H), 7.07-7.03 (m, 1H), 6.49 (s, 1H), 3.56 (ddd, *J* = 10.8, 10.4, 4.4 Hz, 1H), 2.19-2.16 (m, 1H), 1.94-1.89 (m, 1H), 1.65-1.56 (m, 2H), 1.38 (m, 1H), 1.22-0.96 (m, 2H), 0.91 (d, *J* = 6.8 Hz, 3H), 0.87-0.78 (m, 2H), 0.77 (d, *J* = 7.2 Hz, 3H), 0.56 (d, *J* = 6.8 Hz, 3H). ^13^C NMR (100 MHz, CDCl_3_): *δ* = 164.3,163.3,152.2, 152.1,150.7, 140.6, 136.3, 131.1, 130.0, 129.2, 128.4, 125.4, 124.5, 114.8, 102.6, 83.0, 48.1, 42.1, 33.9, 31.5, 25.3, 22.8, 22.1, 21.1, 15.7. MS (ESI): 640.1 [M]^+^, 642.1 [M+2]^+^.

Compound **7d**: 79% yield. [α]_D_^20^ = -44.8 (*c* = 0.5 M, CHCl_3_). mp = 177-179 ^o^C. IR (KBr): 2956, 1779, 1611, 1526, 1475, 1347, 1316, 1257, 1177, 967 cm-^1^. ^1^H NMR (400 MHz, CDCl_3_): *δ* = 8.62 (s, 1H), 8.38-8.35 (m, 2H), 8.01-7.76 (m, 4H), 7.02-6.96 (m, 2H), 6.42 (s, 1H), 5.59 (s, 1H), 3.86 (s, 3H), 3.53 (ddd, *J* = 4.4, 4.4, 4.4 Hz, 1H), 2.16-2.13 (m, 1H),1.61-1.53 (m, 1H), 1.63-1.56 (m, 2H), 1.37-1.35 (m, 1H), 1.20-0.96 (m, 2H), 0.89 (d, *J* = 6.4 Hz, 3H), 0.87-0.77 (m, 2H), 0.74 (d, *J* = 7.2 Hz, 3H), 0.54 (d, *J* = 6.8 Hz, 3H). ^13^C NMR (100 MHz, CDCl_3_): *δ* = 164.3, 163.1,161.7, 152.4, 152.2, 150.6, 136.8, 136.4, 130.0, 124.5, 117.6, 114.7, 114.5, 102.5, 82.9, 55.5 , 48.1, 42.1, 33.9 , 31.5, 25.3, 22. 8, 22.1, 21.1, 15.7. MS (ESI): 670.1 [M]^+^, 672.1 [M+2]^+^.

Compound **7e**: 89% yield. [α]_D_^20^ = -106.6 (*c* = 0.5 M, CHCl_3_). mp = 187-188 ^o^C. IR (KBr): 2955, 1782, 1613, 1596, 1469, 1450, 1316, 1210, 1133, 967 cm-^1^. ^1^H NMR (400 MHz, CDCl_3_): *δ* = 8.24 (s, 1H), 7.90-7.87 (m, 2H), 7.75-7.74 (m, 1H), 7.49-7.45 (m, 3H), 7.12-7.11 (m, 1H), 6.46 (s, 1H), 3.54 (ddd, *J* = 4.4, 4.4, 4.4 Hz, 1H), 2.16-2.13 (m, 1H), 1.92-1.86 (m, 1H), 1.61-1.54 (m, 2H), 1.39-1.32 (m, 1H), 1.23-0.93 (m, 2H), 0.89 (d, *J* = 6.8 Hz, 3H), 0.87-0.81 (m, 2H), 0.74 (d, *J* = 6.0 Hz, 3H), 0.53 (d, *J* = 6.8 Hz, 3H). ^13^C NMR (100 MHz, CDCl_3_): *δ* = 164.5, 155.0, 152. 7, 151.9, 148.5, 140.5, 130.7, 129.1, 128.3, 125.6, 120.9, 114.4, 113.2, 102.7, 82.8, 48.1, 42.0, 33.9, 31.5, 25.2, 22.8, 22.1, 21.0, 15.6. MS (ESI): 585.4 [M]^+^, 587.3 [M+2]^+^.

Compound **7f**: 85% yield. [α]_D_^20^ = -133.60(*c* = 0.5 M, CHCl_3_). mp = 155-157 ^o^C. IR (KBr): 1779, 1609, 1501, 1489, 1323, 1312, 1263, 1126, 998 cm-^1^. ^1^H NMR (400 MHz, CDCl_3_): *δ* = 8.31(s, 1H), 7.88-7.86 (m, 2H), 7.73 (s, 1H), 7.15-7.14 (s, 1H), 7.01-6.99 (m, 2H), 6.68-6.67 (m, 1H), 6.45 (s, 1H), 3.87 (m, 3H), 3.55 (ddd, *J* = 4.0, 4.0, 4.4 Hz, 1H), 2.17-2.14 (m, 1H), 1.92-1.88 (m, 1H), 1.63-1.56 (m, 2H), 1.37-1.38 (m, 1H), 1.24-1.02 (m, 2H), 0.91 (d, *J* = 6.4 Hz, 3H), 0.89-0.83 (m, 2H), 0.79 (d, *J* = 6.4 Hz, 3H), 0.55 (d, *J* = 6.4 Hz, 3H). ^13^C NMR (100 MHz, CDCl_3_): *δ* = 164.5, 161.5, 154.9, 152.8, 151.9, 148.4, 146.9, 139.8, 129.9, 120.9, 117.8, 114.5, 114.2, 113.2, 102.6, 82.7, 55.4, 48.1, 42.0, 33.9, 31.5, 25.2, 22.8, 22.1, 21.1, 15.6. MS (ESI): 615.4 [M]^+^, 617.3 [M+2]^+^.

Compound **7g**: 70% yield. [α]_D_^20^ = -137.6 (*c* = 0.5 M, CHCl_3_). mp = 146-147 ^o^C. IR (KBr): 2955, 1783, 1598, 1565, 1513, 1462, 1316, 1262, 1170, 1134, 967 cm-^1^. ^1^H NMR (400 MHz, CDCl_3_): *δ* = 8.57 (s, 1H), 7.83-7.79 (m, 2H), 7.54-7.51 (m, 2H), 6.34 (s, 1H), 3.48 (ddd, *J* = 4.4, 4.4, 4.4 Hz, 1H), 2.57 (s, 3H), 2.15-2.12 (m, 1H), 1.85-1.81 (m, 1H), 1.64-1.56 (m, 2H), 1.36-1.37 (m, 1H), 1.20-0.95 (m, 2H), 0.92 (d, *J* = 5.6 Hz, 3H), 0.77-0.89 (m, 2H), 0.76 (d, *J* = 2.0Hz, 3H), 0.54 (d, *J* = 6.8 Hz, 3H). ^13^C NMR (100 MHz, CDCl_3_): *δ* = 164.4, 163.5, 152.6, 151.2, 140.2, 138.6, 130.4, 129.8, 129.6, 114.2, 102.5, 82.8, 48.1, 42.1, 33.9, 31.5, 25.2, 22.8, 22.1, 21.1, 15.6, 11.5. MS (ESI): 567.3 [M]^+^, 569.3 [M+2]^+^.

Compound **7h**: 65% yield. [α]_D_^20^ = -128.7 (*c* = 0.5 M, CHCl_3_). mp = 163-164 ^o^C. IR (KBr): 3159, 2955, 1781, 1598, 1565, 1513, 1457, 1316, 1262, 1133, 967 cm-^1^. ^1^H NMR (400 MHz, CDCl_3_): *δ* = 8.37 (s, 1H), 7.77-7.69 (m, 4H), 7.02-6.93 (m, 4H), 6.35 (s, 1H), 3.90 (s, 3H), 3.50 (ddd, *J* = 10.8, 10.4, 4.4 Hz, 1H), 2.14-2.04 (m, 1H), 1.91-1.86 (m, 1H), 1.60-1.54 (m, 2H), 1.32-1.39 (m, 1H), 1.29-0.92 (m, 2H), 0.89 (d, *J* = 6.8 Hz, 3H), 0.88-0.76 ( m, 2H), 0.72 (d, *J* = 9.6 Hz, 3H), 0.53 (d, *J* = 6.4 Hz,3H). ^13^C NMR (100 MHz, CDCl_3_): *δ* = 167.9, 164.6, 164.5 159.2, 152.6, 152.2, 140.1, 131.6, 120.0, 123.4, 116.4, 115.0, 114.4, 102.8, 82.9, 55.7, 48.0, 42.0, 33.9, 31.5, 25.2, 22.7, 22.1, 21.1, 15.7. MS (ESI): 641.2 [M]^+^, 643.2 [M+2]^+^.

Compound **7i**: 75% yield. [α]_D_^20^ = -89.8 (*c* = 0.5 M, CHCl_3_). mp = 151-153 ^o^C. IR (KBr): 3157, 2954, 1783, 1594, 1572, 1514, 1452, 1315, 1289, 1165, 1134, 968 cm-^1^. ^1^H NMR (400 MHz, CDCl_3_): *δ* = 8.33 (s, 1H), 7.79-7.70 (m, 4H), 7.50-7.47 (m, 3H), 7.07-7.05 (m, 2H), 6.38 (s, 1H), 3.51 (ddd, *J* = 4.4, 4.4, 4.4 Hz, 1H), 2.16-2.13 (m, 1H), 1.94-1.90 (m, 1H), 1.61-1.55 (m, 2H), 1.37-1.32 (m, 1H), 1.26-0.94 (m, 2H), 0.91 (d, *J* = 6.4 Hz, 3H), 0.89-0.77 (m, 2H), 0.76 (d, *J* = 9.6 Hz, 3H), 0.56 (d, *J* = 6.8 Hz, 3H). ^13^C NMR (100 MHz, CDCl_3_): *δ* = 168.3, 164.5, 162.4, 152.5, 152.1, 140.7, 131.9, 130.9, 129.1, 128.3, 125.2, 122.7, 116.8, 114.6, 102.8, 83.0, 48.0, 42.0, 33.9, 31.5, 25.3, 22.8, 22.1, 21.1, 15.7. MS (ESI): 611.3 [M]^+^, 613.3 [M+2]^+^.

Compound **7j**: 80% yield. [α]_D_^20^ = -11.9 (*c* = 0.5 M, CHCl_3_). mp = 172-174 ^o^C. IR (KBr): 2953, 1782, 1594, 1565, 1469, 1451, 1323, 1215, 1133, 994, 965 cm-^1^. ^1^H NMR (400 MHz, CDCl_3_): *δ* = 7.94-7.78 (m, 4H), 7.55-7.48 (m, 6H), 6.35 (s, 1H), 3.84 (m, 1H), 2.23-2.22 (m, 1H), 1.69-1.09 (m, 6H), 0.84-0.73 (m, 6H), 0.53 (s, 3H). ^13^C NMR (100 MHz, CDCl_3_): *δ* = 166.4, 164.2, 153.5, 140.5, 139.4, 130.8, 130.5, 129.9, 129.9, 129.3, 129.0, 128.3, 125.6, 103.3, 88.8, 49.4, 47.5, 44.8, 36.6, 27.9, 26.5, 19.5, 18.7, 13.3. MS (ESI): 627.3 [M]^+^, 629.2 [M+2]^+^.

Compound **7k**: 72% yield. [α]_D_^20^ = -11.9 (*c* = 0.5 M, CHCl_3_). mp = 143-144 ^o^C. IR (KBr): 3154, 1780, 1597, 1566, 1513, 1462, 1316, 1260, 1170, 993, 837 cm-^1^. ^1^H NMR (400 MHz, CDCl_3_): *δ* = 8.41 (s, 1H), 7.90-7.74 (m, 4H), 7.04-6.92 (m, 4H), 6.28 (s, 1H), 3.89 (m, 3H), 3.82 (m, 1H), 2.22-2.15 (m, 1H), 1.69-1.06 (m, 6H), 0.78-0.75 (m, 6H), 0.50 (s, 3H). ^13^C NMR (100 MHz, CDCl_3_): *δ* = 168.2, 164.5, 164.4, 159.0, 153.6, 152.4, 139.3, 131.6, 130.1, 123.4, 116.7, 116.3, 115.0, 112.4, 103.4, 88.7, 55.7, 49.4, 47.6, 44.8, 36.6, 27.9, 26.5, 19.5, 18.7, 13.3. MS (ESI): 638.9 [M]^+^, 641.0 [M+2]^+^.

Compound **7l**: 76% yield. [α]_D_^20^ = -37.2 (*c* = 0.5 M, CHCl_3_). mp 131-132 ^o^C. IR (KBr) 3210, 1780, 1603, 1523, 1440, 1421, 1319, 1212, 1134, 993 cm^-1^. ^1^H NMR (400 MHz, CDCl_3_) 10.04 (s, 1H), 8.73 (s, 1H), 7.59-7.04 (m, 2H), 7.14-7.06 (m, 2H), 6.20 (s, 1H), 3.81 (m, 1H), 2.59 (s, 3H), 2.25-2.22 (m, 1H), 1.69-1.09 (m, 6H), 0.78-0.74 (m, 6H), 0.53 (s, 3H). ^13^C NMR (100 MHz, CDCl_3_) 170.4, 164.0, 160.3, 152.9, 151.0, 138.2, 136.3, 133.7, 120.6, 118.1, 115.4, 112.8, 103.1, 88.8, 49.3, 47.6, 44.7, 36.7, 27.9, 26.5, 19.5, 18.7, 13.3, 11.2. HRMS calcd. for C_24_H_27_Br N_4_O_4_S [M]^+^: 546.0936, found 546.0933.
